# Comparing Two Targeted Biopsy Schemes for Detecting Clinically Significant Prostate Cancer in Magnetic Resonance Index Lesions: Two- to Four-Core versus Saturated Transperineal Targeted Biopsy

**DOI:** 10.3390/cancers16132306

**Published:** 2024-06-23

**Authors:** Juan Morote, Nahuel Paesano, Natàlia Picola, Berta Miró, José M. Abascal, Pol Servian, Enrique Trilla, Olga Méndez

**Affiliations:** 1 Department of Urology, Vall d’Hebron University Hospital, 08035 Barcelona, Spain; enrique.trilla@vallhe-bron.cat; 2 Department of Surgery, Universitat Autònoma de Barcelona, 08193 Bellaterra, Spain; npaesa@gmail.com; 3 Research Group in Urology, Vall d’Hebron Research Institute, 08035 Barcelona, Spain; olga.mendez@vhir.org; 4 Clinica Creu Blanca, 08018 Barcelona, Spain; 5 Department of Urology, Bellvitge University Hospital, Hospitalet de Llobregat, 08907 Barcelona, Spain; npicola.bellvitge@gencat.cat; 6 Statistics Unit, Vall d’Hebron Research Institute, 08035 Barcelona, Spain; berta.miro@vhir.org; 7 Department of Urology, Parc de Salut Mar, 08003 Barcelona, Spain; jabascal@psmar.cat; 8 Department of Health Sciences, Universitat Pompeu Fabra, 08003 Barcelona, Spain; 9 Department of Urology, Hospital Germans Trias I Pujol, 08916 Badalona, Spain; pservian.germanstrias@gencat.cat

**Keywords:** prostate cancer, targeted biopsy, index lesion, number of cores

## Abstract

**Simple Summary:**

Targeted biopsies of suspicious lesions detected in magnetic resonance imaging are crucial to discover clinically significant prostate cancer, especially those corresponding to index lesions. However, the optimal scheme for targeted biopsies remains unclear, despite the fact that a two- to four-core scheme is usually recommended. Saturated biopsies of the prostate gland have shown high efficiency in detecting significant PCa. In this article, we report a better efficacy for mapping using a 0.5 mm core biopsy scheme than that using the two- to four-core scheme for detecting clinically significant prostate cancer in magnetic resonance index lesions.

**Abstract:**

Since the optimal scheme for targeted biopsies of magnetic resonance imaging (MRI) suspicious lesions remains unclear, we compare the efficacy of two schemes for these index lesions. A prospective trial was conducted in 1161 men with Prostate Imaging Reporting and Data System v 2.1 3–5 undergoing targeted and 12-core systematic biopsy in four centers between 2021 and 2023. Two- to four-core MRI-transrectal ultrasound fusion-targeted biopsies via the transperineal route were conducted in 900 men in three centers, while a mapping per 0.5 mm core method (saturated scheme) was employed in 261 men biopsied in another center. A propensity-matched 261 paired cases were selected for avoiding confounders other than the targeted biopsy scheme. CsPCa (grade group ≥ 2) was identified in 125 index lesions (41.1%) when the two- to four-core scheme was employed, while in 187 (71.9%) when the saturated biopsy (*p* < 0.001) was used. Insignificant PCa (iPCa) was detected in 18 and 11.1%, respectively (*p* = 0.019). Rates of csPCa and iPCa remained similar in systematic biopsies. CsPCa detected only in systematic biopsies were 5 and 1.5%, respectively (*p* = 0.035) in each group. The saturated scheme for targeted biopsies detected more csPCa and less iPCa than did the two- to four-core scheme in the index lesions. The rate of csPCa detected only in the systematic biopsies decreased when the saturated scheme was employed.

## 1. Introduction

Risk-stratified prostate cancer (PCa) screening, based on serum prostate-specific antigen (PSA) testing and magnetic resonance imaging (MRI) scanning as a follow-up measure, is currently recommended by the European Union [1]. This new paradigm of PCa screening is focused on maximizing the early detection of clinically significant PCa (csPCa), while minimizing the number of prostate biopsies and the over-detection of insignificant prostate cancer (iPCa) [2,3,4,5].

The Prostate Imaging Reporting and Data System (PI-RADS) predicts the risk of csPCa according to the imaging characteristics of lesions detected in MRI scans [6,7,8]. Men with PI-RADS < 3 have a very low probability of csPCa, and consequently, in this case, prostate biopsies can usually be avoided [9,10]. MRI can visualize most lesions containing csPCa, reporting them as PI-RADS 3 to 5, with an intermediate, high, and very high-risk of csPCa, respectively [11]. PI-RADS 3 is considered the gray zone of a PI-RADS score, since the csPCa detection rate is 18.5%, with a 95% confidence interval between 16.6 and 20.3%, but a range between 3.4 and 46.5% [12]. A significant contribution of MRI is the possibility of conducting targeted biopsies for suspicious lesions through MRI-transrectal ultrasound (TRUS) fusion images [13]. However, a small percentage of csPCa are not visualized in MRI scans and are detected only in systematic biopsies, which is the reason why these biopsies are still recommended [14].

The recommendation for changing the approach of prostate biopsies from the classic transrectal to the transperineal route has reduced the infrequent but dangerous infectious complications and the inappropriate use of antibiotics [15,16] accompanying this method. It is known that a prostate biopsy scheme for avoiding the upgrading of csPCa in radical prostatectomy specimens requires a mapping biopsy per 0.5 mm, with additional targeted biopsies of suspicious lesions detected by MRI [17]. However, systematic biopsies complementing targeted biopsies for suspicious lesions causes significant over-detection of iPCa [18].

The currently recommended prostate biopsy scheme suggests obtaining a two- to four-core targeted biopsy of suspicious lesions and a 12-core systematic biopsy [14,19,20,21]. However, the appropriate number of cores obtained from targeted biopsies remains unclear. Two studies have examined the correlation between the PCa grade groups detected in prostate biopsies and those observed in radical prostatectomy specimens, suggesting that obtaining more cores from suspicious lesions reduces the likelihood of upgrading [22,23]. Other studies, conducted in men undergoing transperineal prostate biopsies, suggest the improved detection of csPCa when a saturation scheme for targeted biopsies is employed; however, the results are not uniform [24,25,26,27]. The potential of the index lesion, defined from MRI as the largest lesion with the highest PI-RADS score, for defining the aggressiveness of PCa is known [28,29,30]. However, an appropriate biopsy scheme for the index lesion could minimized unnecessary biopsies from other areas of the prostate gland, as well as systematic biopsies [31]. Mainly, the specifics regarding the differences between the two- to four-core and the saturated scheme for targeted biopsies is the rationale of mapping each 0.5 mm, which has been shown to be the most appropriate scheme for avoiding upgrading in radical prostatectomy specimens [13,22,23,24,25,26,27].

We hypothesize than the saturated scheme for targeted biopsies detects more csPCa in the index lesions than does the recommended two- to four-core scheme. The objectives of this study are to compare the rates of csPCa and iPCa detection in both targeted biopsy schemes and to assess the rates of csPCa detected only in systematic biopsies.

## 2. Materials and Methods

### 2.1. Design, Setting, and Participants

A prospective non-randomized trial including 1161 consecutive men suspected of having PCa, based on a serum PSA > 3.0 ng/mL, was conducted in four centers participa-ting in the csPCa opportunistic early detection program of Catalonia (Spain) between 1 January 2021 and 30 June 2023. All included men exhibited PI-RADS v 2.1 lesions 3 to 5 in pre-biopsy multiparametric MRI (mpMRI), undergoing targeted biopsy of suspicious lesions and 12-core systematic biopsy. Three participant centers, always obtaining two- to four-cores from each suspicious lesion, reported 900 cases, while 261 cases were reported in another center, where a mapping per 0.5 mm core scheme was always employed. Due to the possible influence on csPCa detection, men undergoing treatment with 5-alpha reductase inhibitors and those with prior history of PCa, atypical small acinar proliferation, or multifocal high-grade prostatic intraepithelial neoplasia were not included in this study.

### 2.2. Diagnostic Approach for csPCa Detection

MpMRI using a 3 Tesla scan was performed in each participant center using a pelvic phased-array surface coil. The acquisition protocol included T2-weighted imaging (T2W), diffusion-weighted imaging (DWI), and dynamic contrast-enhanced (DCE) imaging, according to the guidelines of the European Society of Urogenital Radiology [6]. MpMRI exams were reported by local expert radiologists using the PI-RADS v 2.1 [8]. Local experienced operators performed prostate biopsies using the Koelis Trinity^®^ hands-free MRI/TUS prostate biopsy system (Koelis Inc., Grenoble, France) in the centers conducting targeted biopsies with the two- to four-core scheme, while the Artemis^®^ hands-free MRI/TUS prostate biopsy system (Eigen Inc., Grass Valley, CA, USA) was used in the center performing the mapping per 0.5 mm core scheme. Experienced local uropathologists exa-mined the biopsy material in each pathology department, reporting PCa using the International Society of Urologic Pathology grade group (GG) classification. CsPCa was considered when the GG was ≥2 [32]. Baseline characteristics of the study cohort are summarized in Table 1.

### 2.3. Variables in the Study and Outcome Variables

Age (years), first degree PCa family history (no vs. yes), type of prostate biopsy (initial vs. repeated), serum PSA (ng/mL), DRE (normal vs. suspicious), MRI-prostate volume (mL), PI-RADS score v 2.1 (3 to 5), size and localization of the index lesion, targeted biopsy scheme (two- to four-core vs. mapping per 0.5-core) were predictive variables in the study. Outcome variables were csPCa and iPCa detection.

### 2.4. Statistical Analysis

Statistical analysis was conducted after harmonization of the anonymized datasets. Data were prospectively collected and reported according to the Standards of Reporting for MRI-Targeted Biopsy Studies (START) to describe the study population [33]. Quantitative variables are described using medians and interquartile ranges (IQR: 25th–75th percentile), while qualitative variables are described using numbers and percentages. Quantitative variables were compared between groups using the Mann–Whitney U test. Qua-litative variables were compared between groups using Pearson’s chi-square test. Relative risk (RR) of csPCa and 95% confidence intervals (CI) were assessed. A logistic regression analysis conducted for detecting independent predictive variables of csPCa, in addition to the targeted biopsy scheme, showed that PI-RADS score, prostate volume, age, serum PSA, location, and size of the index lesion were independent predictors of csPCa and were potential confounders for csPCa detection (Table 2).

A randomized 1:1 matched group from all predictive variables was selected using the R package matching v 4.10, a multivariate and propensity score matching software with automated balance optimization (R Foundation for Statistical Computing, Vienna, Austria). In a subset of 522 men, the two- to four-core scheme was employed, and the mapping per 0.5 mm core scheme was used in the other 261 men. Table 3 shows the sui-tability of this matched paired group for analysis, since all baseline confounder characte-ristics for csPCa detection were equally distributed in both study subsets. A *p* value of <0.05 was considered statistically significant. The data were analyzed using the Statistical Package for the Social Sciences (version 29.0; IBM Corp., Armonk, NY, USA).

## 3. Results

### 3.1. Baseline Characteristics of the Study Population

The baseline characteristics of the propensity-matched group comprising 522 men, including 261 men who underwent targeted biopsies employing the two- to four-core scheme and 261 who underwent the mapping per 0.5 mm core scheme, were similar except in regards to the median number of cores utilized in each targeted biopsy scheme, which were 2 (IQR 1–2) and 9 (IQR 5–12), respectively (*p* = 0.016). The detection rate of PCa in the index lesion and the 12-core systematic biopsies, according to the employed scheme, were 63.6 and 82.7%, respectively (*p* < 0.001), with 45.8 and 71.6% csPCa, and 18.1 and 11.1% iPCa, respectively (*p* = 0.012), Table 3.

### 3.2. CsPCa Detection in the Index Lesions according to the PI-RADS Score and the Biopsy Scheme

**Figure 1:**
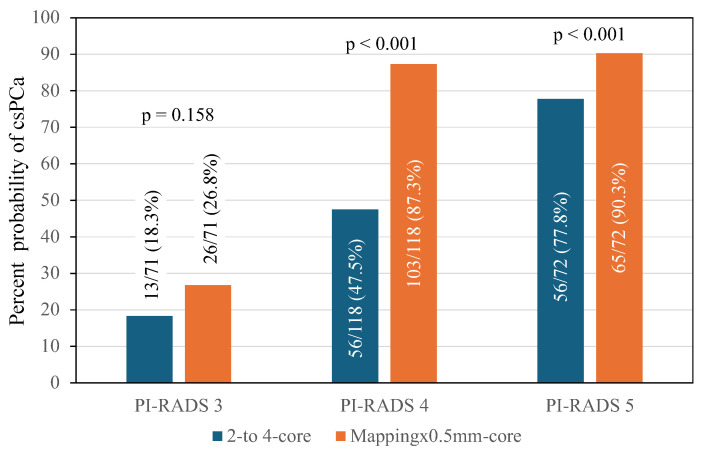
CsPCa detection in the index lesion targeted biopsies, according to the employed two- to four-core and mapping × 0.5 mm core schemes and the PI-RADS score.

Among 142 men with a PI-RADS score of 3, csPCa was identified in 13 of 71 (18.3%) index lesions biopsied using the two- to four-core scheme, while in 26 of 71 (26.8%) when the mapping per 0.5 mm core scheme was employed, with an RR of 1.298 (95% CI 0.824–2.045), *p* = 0.158. Among 236 men with a PI-RADS score of 4, these rates were 47.4% and 87.3%, respectively, with an RR of 2.286 (95% CI 1.802–2.900), *p* = 0.001. Among 144 men with a PI-RADS score of 5, the rates were 77.8% and 90.3%, respectively, with an RR of 1.503 (95% CI 1.079–2.094), *p* < 0.001 (Figure 1).

### 3.3. Detection of csPCa and iPCa in the Index Lesions, According to the Employed Biopsy Scheme and Those Detected in Systematic Biopsies

**Figure 2:**
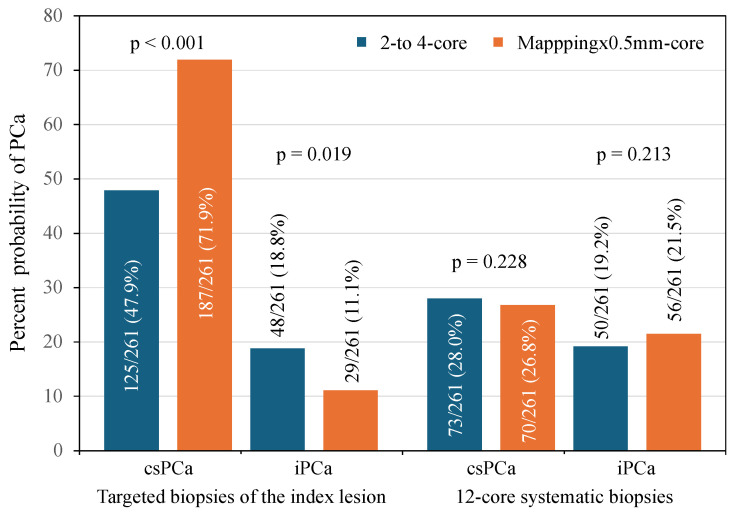
Detection of csPCa and iPCa in the index lesion targeted biopsies and the 12-core syste-matic biopsies, according to the two- to four-core and mapping × 0.5 mm core schemes employed in the targeted biopsies.

Figure 2 summarizes the csPCa and iPCa detection in the targeted biopsies of the index lesions and systematic biopsies, according to the employed targeted biopsy scheme. CsPCa detection in the index lesions was 47.9% when two- to four-core scheme was applied, while 71.9% when mapping per 0.5 mm core scheme was used, with an RR of 1.616 (95% CI 1.366–1.913), *p* < 0.001. IPCa detected in the index lesions were 18.8 and 11.1%, respectively, with an RR of 0.760 (9% CI 0.624–0.925), *p* = 0.006. The csPCa detection rates in systematic biopsies, according to the biopsy scheme employed in the targeted biopsies, corresponded to 28.0%, when the two- to four-core scheme was used, and 26.8%, when the mapping per 0.5 mm core scheme was employed, with an RR of 0.829 (95% CI 0.789–1.121), *p* = 0.228. The IPCa detection rates in systematic biopsies were 19.2% and 21.5%, respectively, with an RR of 1.191 (95% CI 0.940–1.909), *p* = 0.187.

**Figure 3:**
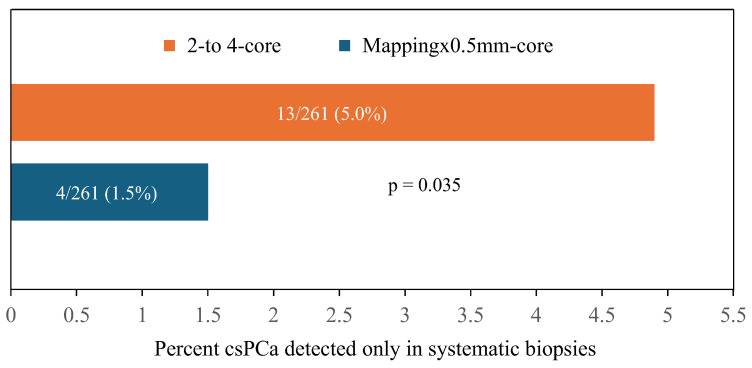
CsPCa detection in only the 12-core systematic biopsies, according to the two- to four-core and mapping × 0.5 mm core schemes employed in the targeted biopsies.

CsPCa was detected only in 12-core systematic biopsies in 5.0% of cases when the two- to four-core scheme was applied in the targeted biopsies, while it decreased to 1.5% when the mapping per 0.5 mm core scheme was employed, with an RR of 0.642 (95% CI 0.486–0.848), *p* = 0.213 (Figure 3).

## 4. Discussion

The analysis of this prospective and multicenter trial was conducted in a propensity-paired matched group to avoid the influence of existing confounder variables for csPCa detection other than the targeted biopsy scheme. Our main findings were, (i) the csPCa detection in the index lesions improved when the saturated mapping per 0.5 mm core targeted biopsy scheme was employed, compared with the results for the currently recommended two- to four-core scheme; (ii) this improvement was observed in all PI-RADS categories, although it was significant only in categories 4 and 5; (iii) the iPCa detection rates in the index lesions decreased when the saturated scheme was employed, compared with the results for the two- to four-core scheme; (iv) the percentage of csPCa detected only in systematic biopsies decreased when the saturated scheme was employed compared to the results for the two- to four-core scheme.

In 2013, the Ginsburg Study Group first proposed obtaining two to four cores in targeted biopsies of suspicious lesions in MRI-TRUS fusion prostate biopsies conducted via the transperineal route. This group also proposed at least 4-core biopsies of the anterior, mid, posterior, and basal sectors of the right and left prostate lobules, resulting in 24 cores for prostate glands of less than 30 mL, 32 cores for those of 30 to 50 mL, and 38 additional cores for prostate glands of more than 50 mL [17]. In 2016, Radtke et al. observed that the Ginsburg approach was able to identify 97% of csPCa detected in 120 radical prostatectomy specimens, while only 79% of them were identified in systematic biopsies, and 88% were found in targeted biopsies alone [22]. In 2018, Calio et al. examined 122 men who underwent targeted MRI-TRUS index lesion biopsies employing a two-core scheme and 86 men who underwent a mapping per 0.6 mm core scheme. Additionally, all men underwent a 12-core systematic biopsy. The authors observed a surgical specimen upgrading of 40.9% with systematic biopsies, 23.6% with two-core targeted biopsies, and 13.8% with mapping per 0.6 mm core targeted biopsies [23]. Many studies have focused their attention on the findings in the index lesions, due to its high predictive power for defining PCa aggressiveness [28,29,30].

It is difficult to compare our results with those observed in previous reports due to the variability of study designs. In 2020, Hansen et al. analyzed 487 men who underwent transperineal MRI-TRUS prostate biopsies following the Ginsburg approach. Targeted biopsies of suspicious lesions using the two-core scheme only detected 67% of csPCa. In contrast, extended targeted biopsies obtaining two additional cores from sectors adjacent to the index lesion detected 76% of csPCa, and targeted biopsies using a six-core scheme detected 91% of csPCa [24]. This study suggested that obtaining more cores from targeted biopsies of the index lesion improved csPCa detection. In 2021, Tschirdewahn et al. reported a study conducted in 213 men who underwent targeted biopsies of suspicious MRI lesions, employing the Ginsburg approach, between 2016 and 2018. Targeted biopsies were collected using a four-core scheme. Targeted biopsies involved collecting 4 cores from suspicious lesions and additional cores from adjacent sectors, and systematic biopsies obtained 9 to 10 cores. Targeted saturated biopsies detected 99% of 134 csPCa, while non-saturated targeted biopsies detected 87%, and systematic biopsies detected 82% [25]. This study also confirms that greater core obtention in targeted biopsies increases csPCa detection.

In 2023, Cetin et al. reported an analysis of 167 men in whom targeted biopsies obtained two cores from the center of the suspicious lesions and two additional cores from the periphery. One central core identified 65.6% of csPCa, two central cores identified 92.2%, two central cores and one peripheral core identified 96.9%, and two central cores and two peripheral cores identified 100% of csPCa. These authors concluded that the two central cores scheme was sufficient to detect the vast majority of csPCa because the diffe-rence between two and four cores was non-significant [25].

Finally, in 2023, Sanner et al. reported a prospective single-center trial randomizing 170 men who underwent transperineal targeted biopsy of suspicious lesions employing a four-core scheme compared to targeted saturated biopsies using a nine-core scheme. Both schemes were complemented with a 24-core systematic biopsy. Targeted biopsies detected 91.7% of csPCa, while targeted saturated biopsies detected 100%. Because this difference was non-significant, the authors concluded that csPCa detection did not differ between targeted and saturated targeted biopsies [27]. The results of this prospective and randomized trial contradict the previous retrospective study reported by the same authors [24]. A recently reported study has noted the importance of only biopsying the index lesion with an appropriate scheme for avoiding targeted biopsies of secondary lesions and systematic biopsies [31]. Additionally, our results suggest that saturated targeted biopsies reduce the rate of csPCa detected only in systematic biopsies. This finding requires confirmation in additional studies.

Our study is limited by its non-randomized design, although the propensity-matched paired group selection minimized the effect of confounder variables for csPCa detection. The utilization of different devices for MRI-TRUS biopsies and the level of experience of the surgeon performing the two different types of prostate biopsies can influence the results, as can the inter-variability between pathologists. The csPCa outcome va-riable detected in prostate biopsies does not represent the true csPCa detected in the radical prostatectomy specimens. Since we decided to conduct this study using the index lesions, a limitation exists in regards to recommending the saturated targeted biopsy scheme for all suspicious lesions. A limitation exists for recommending a prostate biopsy approach based only on a saturated targeted scheme of the index lesion, since the current knowledge suggests the importance of perilesional biopsies for avoiding ipsilateral systematic biopsies [34,35,36,37].

One of the strengths of this study is that it is conducted using matched pairs of men suspected of having PCa in order to avoid the influence of confounders for csPCa detection. One of the study’s weakness is that it is retrospective, with a non-randomized design. However, new evidence is generated for recommending saturated targeted biopsies, possibly without systematic biopsies, if perilesional biopsies are obtained. Well-designed prospective and multicenter randomized trials identifying the most effective and least aggressive prostate biopsy scheme for transperineal prostate biopsy are needed for maxi-mizing the detection of csPCa, minimizing the over-detection of iPCa, and reducing prostate biopsy site effects.

## 5. Conclusions

Saturated targeted biopsies, using a mapping per 0.5 mm core scheme, were able to detect more csPCa and less iPCa than the recommended two- to four-core scheme in the index lesions. This advantage was particularly notable in individuals with PI-RADS scores above 3. The rate of csPCa detected only in 12-core systematic biopsies decreased when the mapping per 0.5 mm core scheme for targeted biopsies was employed.

## Figures and Tables

**Table 1:** Characteristics of cohort study.

Characteristic	Measurement
Number of men	1161
Median age, years (IQR)	67 (61–73)
Median serum PSA, ng/mL (IQR)	6.7 (5.1–9.9)
Abnormal DRE, *n* (%)	200 (25.8)
Median prostate volume, ml (IQR)	50 (36–70)
Prior negative prostate biopsy, *n* (%)	403 (34.7)
Family history of PCa, *n* (%)	96 (8.3%)
Median suspicious lesions, *n* (IQR)	1 (1–2)
Median length of suspicious lesions, mm (IQR)	11 (5–17)
Localization of the index lesion, *n* (%)	
Peripheral zone	835 (71.9)
Central-transition zone	278 (23.9)
Anterior zone	48 (4.2)
PI-RADS score of the index lesion, *n* (%)	
3	260 (22.4)
4	587 (50.4)
5	315 (27.1)
Median cores obtained in targeted biopsy, *n* (IQR) index lesion, *n* (IQR)	3 (1–7)
Overall PCa detection, *n* (%)	815 (70.2)
csPCa	601 (51.8)
iPCa	241 (18.4)

IQR = interquartile range; *n* = number; PCa = prostate cancer; csPCa = clinically significant PCa; iPCa = insignificant PCa.

**Table 2:** Multivariate analysis searching confounder variables for csPCa detection in the index lesion, in addition to the targeted biopsy scheme employed.

Predictive Variable	Odds Ratio (95% CI)	*p* Value
Age, Ref. year	1.061 (1.036–1.086)	<0.001
Serum PSA, Ref. ng/mL	1.062 (1.027–1.099)	<0.001
DRE, Ref. normal	1.154 (0.775–1.718)	=0.481
Type of biopsy, Ref. initial	0.960 (0.695–1.429)	=0.841
PCa family history, Ref. no	1.258 (0.749–2.111)	=0.386
Prostate volume, Ref. mL	0.972 (0.065–0.979)	<0.001
Number of suspicious lesions, Ref. one	1.387 (0.863–2.231)	=0.177
PI-RADS score of the index lesion, Ref. 3	2.718 (2.032–3.365)	<0.001
Size of the index lesion. Ref. mm	1.071 (1.030–1.113)	<0.001
Localization of the index lesion, Ref. peripheral zone	0.611 (0.438–0.852)	=0.004
Targeted prostate biopsy scheme, Ref. 2- to 4-core	2.137 (1.869–3.439)	<0.001

CI = confidence interval; PSA = prostate specific antigen; DRE = digital rectal examination; PCa = prostate cancer; PI-RADS = Prostate Imaging Reporting and Data System.

**Table 3:** Baseline characteristics of the paired matched study group and a comparative analysis between them, according to biopsy scheme applied for targeted biopsy of the index lesions.

Baseline Characteristic	Biopsy Scheme of the Index Lesion		*p* Value
	Two- to Four-Core	Mapping × 0.5 mm Core	
Number of men, *n* (%)	261 (50.0)	261 (50.0)	-
Median age, years (IQR)	67 (61–73)	67 (61–73)	=1.000
Median serum PSA, ng/mL (IQR)	6.7 (5.0–9.6)	6.7 (5.0–9.6)	=1.000
Abnormal DRE, *n* (%)	65 (24.9)	67 (25.7)	=0.678
Median prostate volume, ml (IQR)	45 (33–62)	45 (33–62)	=1.000
Prior negative prostate biopsy, *n* (%)	63 (24.1)	68 (26.0)	=0.357
Family history of PCa, *n* (%)	30 (11.5%)	28 (10.7)	=0.419
Median suspicious lesions, *n* (IQR)	1 (1–2)	1 (1–2)	=1.000
Median length of the index lesion, mm (IQR)	12 (9–18)	12 (9–18)	=1.000
Index lesion localization, *n* (%)			
Peripheral zone	179 (68.6)	179 (68.6)	=1.000
Central/transition zone	62 (23.8)	62(23.8)	=1.000
Anterior zone	20 (7.7)	20 (7.7)	=1.000
PI-RADS score of index lesion, *n* (%)			
3	71 (27.2)	71 (27.2)	=1.000
4	118 (45.2)	118 (45.2)	=1.000
5	72 (27.6)	72 (27.6)	=1.000
Median number of cores in the index lesion, *n* (IQR) obtained, *n* (IQR)	2 (1–3)	9 (5–12)	=0.016
Overall PCa detection, *n* (%)	328 (63.9)	216 (82.7)	<0.001
csPCa, *n* (%)	235 (45.8)	187 (71.6)	<0.001
iPCa, n (%)	93 (18.1)	29 (11.1)	=0.012

IQR = interquartile range; *n* = number; PI-RADS = Prostate Imaging Reporting and Data System; PCa = prostate cancer; csPCa = clinically significant PCa; iPCa = insignificant PCa.

## Data Availability

Datasets from this study are available upon request from the corresponding author.

## References

[1] Van Poppel H., Roobol M.J., Chandran A. (2023). Early Detection of Prostate Cancer in the European Union: Combining Forces with PRAISE-U. Eur. Urol..

[2] Van Poppel H., Hogenhout R., Albers P., van den Bergh R.C.N., Barentsz J.O., Roobol M.J. (2020). Early Detection of Prostate Cancer in 2020 and Beyond: Facts and recommendations for the European Union and the European Commission. Eur. Urol..

[3] Van Poppel H., Hogenhout R., Albers P., van den Bergh R.C.N., Barentsz J.O., Roobol M.J. (2021). A European Model for an Organised Risk-stratified Early Detection Programme for Prostate Cancer. Eur. Urol. Oncol..

[4] Van Poppel H., Roobol M.J., Chapple C.R., Catto J.W.F., N’Dow J., Sønksen J., Stenzl A., Wirth M. (2021). Prostate-specific Antigen Testing as Part of a Risk-Adapted Early Detection Strategy for Prostate Cancer: European Association of Urology Position and Recommendations for 2021. Eur. Urol..

[5] Van Poppel H., Albreht T., Basu P., Hogenhout R., Collen S., Roobol M. (2022). Serum PSA-based early detection of prostate cancer in Europe and globally: Past, present and future. Nat. Rev. Urol..

[6] Barentsz J.O., Richenberg J., Clements R., Choyke P., Verma S., Villeirs G., Rouviere O., Logager V., Futterer J.J. (2012). ESUR prostate MR guidelines 2012. Eur. Radiol..

[7] Weinreb J.C., Barentsz J.O., Choyke P.L., Cornud F., Haider M.A., Macura K.J., Margolis D., Schnall M.D., Shtern F., Tempany C.M. (2016). PI-RADS Prostate Imaging–Reporting and Data System: 2015, Version 2. Eur. Urol..

[8] Turkbey B., Rosenkrantz A.B., Haider M.A., Padhani A.R., Villeirs G., Macura K.J., Tempany C.M., Choyke P.L., Cornud F., Margolis D.J. (2019). Prostate Imaging Reporting and Data System Version 2.1: 2019 Update of Prostate Imaging Reporting and Data System Version 2. Eur. Urol..

[9] Moldovan P.C., Van den Broeck T., Sylvester R., Marconi L., Bellmunt J., van den Bergh R.C., Bolla M., Briers E., Cumberbatch M.G., Fossati N. (2017). What Is the Negative Predictive Value of Multiparametric Magnetic Resonance Imaging in Excluding Prostate Cancer at Biopsy? A Systematic Review and Meta-analysis from the European Association of Urology Prostate Cancer Guidelines Panel. Eur. Urol..

[10] Wagaskar V.G., Levy M., Ratnani P., Moody K., Garcia M., Pedraza A.M., Parekh S., Pandav K., Shukla B., Prasad S. (2021). Clinical Utility of Negative Multiparametric Magnetic Resonance Imaging in the Diagnosis of Prostate Cancer and Clinically Significant Prostate Cancer. Eur. Urol. Open Sci..

[11] Oerther B., Engel H., Bamberg F., Sigle A., Gratzke C., Benndorf M. (2022). Cancer detection rates of the PI-RADSv2.1 assessment categories: Systematic review and meta-analysis on lesion level and patient level. Prostate Cancer Prostatic Dis..

[12] Maggi M., Panebianco V., Mosca A., Salciccia S., Gentilucci A., Di Pierro G., Busetto G.M., Barchetti G., Campa R., Sperduti I. (2020). Prostate Imaging Reporting and Data System 3 Category Cases at Multiparametric Magnetic Resonance for Prostate Cancer: A Systematic Review and Meta-analysis. Eur. Urol. Focus.

[13] Ahmed H.U., El-Shater Bosaily A., Brown L.C., Gabe R., Kaplan R., Parmar M.K., Collaco-Moraes Y., Ward K., Hindley R.G., Freeman A. (2017). Diagnostic accuracy of multi-parametric MRI and TRUS biopsy in prostate cancer (PROMIS): A paired validating confirmatory study. Lancet.

[14] Drost F.H., Osses D., Nieboer D., Bangma C.H., Steyerberg E.W., Roobol M.J., Schoots I.G. (2020). Prostate Magnetic Resonance Imaging, with or Without Magnetic Resonance Imaging-targeted Biopsy, and Systematic Biopsy for Detecting Prostate Cancer: A Cochrane Systematic Review and Meta-analysis. Eur. Urol..

[15] Hu J.C., Assel M., Allaf M.E., Ehdaie B., Vickers A.J., Cohen A.J., Ristau B.T., Green D.A., Han M., Rezaee M.E. (2024). Transperineal Versus Transrectal Magnetic Resonance Imaging-targeted and Systematic Prostate Biopsy to Prevent Infectious Complications: The PREVENT Randomized Trial. Eur. Urol..

[16] Connor M.J., Gorin M.A., Eldred-Evans D., Bass E.J., Desai A., Dudderidge T., Winkler M., Ahmed H.U. (2023). Landmarks in the evolution of prostate biopsy. Nat. Rev. Urol..

[17] Kuru T.H., Wadhwa K., Chang R.T., Echeverria L.M., Roethke M., Polson A., Rottenberg G., Koo B., Lawrence E.M., Seidenader J. (2013). Definitions of terms, processes and a minimum dataset for transperineal prostate biopsies: A standardization approach of the Ginsburg Study Group for Enhanced Prostate Diagnostics. BJU Int..

[18] Hugosson J., Månsson M., Wallström J., Axcrona U., Carlsson S.V., Egevad L., Geterud K., Khatami A., Kohestani K., Pihl C.G. (2022). Prostate Cancer Screening with PSA and MRI Followed by Targeted Biopsy Only. N. Engl. J. Med..

[19] Gandaglia G., Pellegrino A., Montorsi F., Briganti A. (2022). Prostate Cancer: Is There Still a Role for Systematic Biopsies? Yes. Eur. Urol. Open Sci..

[20] (2024). EAU-EANM-ESTRO-ESUR-ISUP-SIOG Guidelines on Prostate. Cancer. http://uroweb.org/guidelines/compilations-of-all-guidelines/.

[21] Wei J.T., Barocas D., Carlsson S., Coakley F., Eggener S., Etzioni R., Fine S.W., Han M., Kim S.K., Kirkby E. (2023). Early Detection of Prostate Cancer: AUA/SUO Guideline Part II: Considerations for a Prostate Biopsy. J. Urol..

[22] Radtke J.P., Schwab C., Wolf M.B., Freitag M.T., Alt C.D., Kesch C., Popeneciu I.V., Huettenbrink C., Gasch C., Klein T. (2016). Multiparametric Magnetic Resonance Imaging (MRI) and MRI-Transrectal Ultrasound Fusion Biopsy for Index Tumor Detection: Correlation with Radical Prostatectomy Specimen. Eur. Urol..

[23] Calio B.P., Sidana A., Sugano D., Gaur S., Maruf M., Jain A.L., Merino M.J., Choyke P.L., Wood B.J., Pinto P.A. (2018). Risk of Upgrading from Prostate Biopsy to Radical Prostatectomy Pathology-Does Saturation Biopsy of Index Lesion during Multiparametric Magnetic Resonance Imaging-Transrectal Ultrasound Fusion Biopsy Help. J. Urol..

[24] Hansen N.L., Barrett T., Lloyd T., Warren A., Samel C., Bratt O., Kastner C. (2020). Optimising the number of cores for magnetic resonance imaging-guided targeted and systematic transperineal prostate biopsy. BJU Int..

[25] Tschirdewahn S., Wiesenfarth M., Bonekamp D., Püllen L., Reis H., Panic A., Kesch C., Darr C., Heß J., Giganti F. (2021). Detection of Significant Prostate Cancer Using Target Saturation in Transperineal Magnetic Resonance Imaging/Transrectal Ultrasonography-fusion Biopsy. Eur. Urol. Focus.

[26] Cetin S., Huseyinli A., Koparal M.Y., Bulut E.C., Ucar M., Gonul I.I., Sozen S. (2023). How many cores should be taken from each region of interest when performing a targeted transrectal prostate biopsy. Prostate Int..

[27] Saner Y.M., Wiesenfarth M., Weru V., Ladyzhensky B., Tschirdewahn S., Püllen L., Bonekamp D., Reis H., Krafft U., Heß J. (2023). Detection of Clinically Significant Prostate Cancer Using Targeted Biopsy with Four Cores Versus Target Saturation Biopsy with Nine Cores in Transperineal Prostate Fusion Biopsy: A Prospective Randomized Trial. Eur. Uro.l Oncol..

[28] Ahmed H.U. (2009). The index lesion and the origin of prostate cancer. N. Engl. J. Med..

[29] Valerio M., Anele C., Freeman A., Jameson C., Singh P.B., Hu Y., Emberton M., Ahmed H.U. (2015). Identifying the index lesion with template prostate mapping biopsies. J. Urol..

[30] Russo F., Regge D., Armando E., Giannini V., Vignati A., Mazzetti S., Manfredi M., Bollito E., Correale L., Porpiglia F. (2016). Detection of prostate cancer index lesions with multiparametric magnetic resonance imaging (mp-MRI) using whole-mount histological sections as the reference standard. BJU Int..

[31] Paesano N., Catalá V., Tcholakian L., Alomar X., Barranco M., Trilla E., Morote J. (2024). The effectiveness of mapping-targeted biopsies on the index lesion in transperineal prostate biopsies. Int. Braz. J. Urol..

[32] Epstein J.I., Egevad L., Amin M.B., Delahunt B., Srigley J.R., Humphrey P.A., Grading C. (2016). The 2014 International Society of Urological Pathology (ISUP) Consensus Conference on Gleason Grading of Prostatic Carcinoma: Definition of Grading Patterns and Proposal for a New Grading System. Am. J. Surg Pathol..

[33] Moore C.M., Kasivisvanathan V., Eggener S., Emberton M., Fütterer J.J., Gill I.S., Grubb Iii R.L., Hadaschik B., Klotz L., Margolis D.J. (2013). Standards of reporting for MRI-targeted biopsy studies (START) of the prostate: Recommendations from an International Working Group. Eur. Urol..

[34] Raman A.G., Sarma K.V., Raman S.S., Priester A.M., Mirak S.A., Riskin-Jones H.H., Dhinagar N., Speier W., Felker E., Sisk A.E. (2021). Optimizing Spatial Biopsy Sampling for the Detection of Prostate Cancer. J. Urol..

[35] Brisbane W.G., Priester A.M., Ballon J., Kwan L., Delfin M.K., Felker E.R., Sisk A.E., Hu J.C., Marks L.S. (2022). Targeted Prostate Biopsy: Umbra, Penumbra, and Value of Perilesional Sampling. Eur. Urol..

[36] Tomioka M., Seike K., Uno H., Asano N., Watanabe H., Tomioka-Inagawa R., Kawase M., Kato D., Takai M., Iinuma K. (2023). Perilesional Targeted Biopsy Combined with MRI-TRUS Image Fusion-Guided Targeted Prostate Biopsy: An Analysis According to PI-RADS Scores. Diagnostics.

[37] Lombardo R., Tema G., Nacchia A., Mancini E., Franco S., Zammitti F., Franco A., Cash H., Gravina C., Guidotti A. (2023). Role of Perilesional Sampling of Patients Undergoing Fusion Prostate Biopsies. Life.

